# Clinical Evaluation of Diabetic Foot Ulcers Using the SINBAD (Site, Ischemia, Neuropathy, Bacterial Infection, Area, and Depth) Scoring System: A Prospective Study

**DOI:** 10.7759/cureus.89044

**Published:** 2025-07-30

**Authors:** Hrudaya Charan Kunda, Madhu A Yadav, Tanissha Sanjay Raj Kalpana, Dhrushti Premal Patel, Sivanandhini Sivanoli, Naman Dimpal Shah, Shahista Shahista, Jitenkumar Panchal

**Affiliations:** 1 General Surgery, People's Education Society (PES) University Institute of Medical Sciences and Research, Bengaluru, IND; 2 Anesthesia and Critical Care, Sri Padmavathi Children's Heart Center, Tirupati, IND; 3 Surgery, Devarajan Medical Centre, Chennai, IND; 4 Surgery, Grodno State Medical University, Grodno, BLR; 5 Surgery, Sri Venkateshwaraa Medical College, Pondicherry, IND; 6 Surgery, Privolzhsky Research Medical University, Nizhny Novgorod, RUS; 7 Surgery, Krishnanagar Institute of Medical Science (KIMS), Krishnanagar, IND

**Keywords:** amputation, diabetic foot ulcer, healing, limb salvage, sinbad ulcer scoring, ulcer scoring

## Abstract

Background

Diabetic foot ulcers (DFUs) are a serious complication of type 2 diabetes mellitus (T2DM), leading to significant morbidity, prolonged hospitalization, and risk of limb amputation. The SINBAD (Site, Ischemia, Neuropathy, Bacterial infection, Area, and Depth) scoring system evaluates ulcer severity through six clinical parameters and has been proposed as a valuable tool for predicting outcomes. This prospective study aimed to assess the clinical utility of the SINBAD scoring system in predicting surgical outcomes, including ulcer healing, limb salvage, and amputation rates in patients with T2DM presenting with DFUs.

Materials and methods

A total of 130 T2DM patients with clinically confirmed DFUs were enrolled over one year at a tertiary care center. Ulcers were scored using SINBAD parameters: site, ischemia, neuropathy, bacterial infection, area, and depth. Patients received individualized surgical management based on SINBAD scores. Follow-up was conducted weekly for one month and then monthly for six months. Primary outcomes included ulcer healing, limb salvage, and amputation (minor and major).

Results

The mean age was 62.65±12.5 years, with a male predominance (67.7%). The forefoot was the most common site of ulcers (86.1%), with ischemia present in 35.4%, neuropathy in 65.4%, and bacterial infection in 91.5%. Most ulcers (95.4%) were larger than 1 cm². SINBAD scores of 3 were most common (55.4%). Healing rates were highest in patients with SINBAD scores of 1 (90%), while major amputations occurred predominantly in scores 5 and 6 (75% and 100%, respectively). The association between SINBAD scores and clinical outcomes was statistically significant (p<0.05).

Conclusion

The SINBAD scoring system reliably stratifies DFU severity and predicts healing and amputation risks, supporting its use as a practical tool in clinical decision-making to improve patient outcomes.

## Introduction

Type 2 diabetes mellitus (T2DM) is a metabolic disorder characterized by insulin resistance and a deficiency in insulin secretion, leading to elevated glucose concentrations in the bloodstream. This condition constitutes the predominant form of diabetes globally and is linked to various microvascular and macrovascular complications [1]. The global prevalence of diabetes experienced a significant increase, impacting more than 537 million adults in 2021. Projections indicate this number may escalate to 783 million by 2045 [2].

Diabetic foot ulcer (DFU) is one of the devastating complications of T2DM, imposing significant morbidity and mortality and also causing an economic burden to the patient [3]. DFU leads to extended hospital stays, chronic wounds, and, in severe cases, lower limb amputation [4]. Early recognition and prompt management of DFUs are paramount to reducing these detrimental outcomes and improving patient prognosis [5].

The complex etiology of DFU involves peripheral neuropathy, vascular insufficiency, repetitive trauma, and compromised immunity, contributing collectively to ulcer development and delayed wound healing [6]. Due to the wide range of etiological causes, accurate clinical evaluation and risk stratification are needed for effective management and planning [7]. Various scoring systems have been proposed to classify the DFU severity and outcome assessment, aiding in tailored therapeutic strategies [8].

The SINBAD (Site, Ischemia, Neuropathy, Bacterial infection, Area, and Depth) scoring system is a validated, practical tool designed to assess DFU [9]. It systematically evaluates essential clinical parameters that influence wound healing and the potential for limb salvage. The SINBAD score also predicts wound healing, with scores ranging between 2 and 3, and a score of 3 or higher is at a greater risk of poor outcomes, including limb amputation [10]. The SINBAD severity score cut-off for predicting healing or amputation is 3, and this value exhibits good predictability and reproducibility, having been validated in earlier studies [11]. Its clinical applicability and ease of use have made it a valuable asset in routine practice, enabling healthcare providers to identify patients at high risk for poor outcomes promptly. Despite extensive research on DFU management, variability in outcomes remains, underscoring the importance of standardized clinical assessment tools. The current prospective study aims to evaluate the clinical utility of the SINBAD scoring system in predicting surgical outcomes among patients presenting with DFUs. This research aims to identify critical predictors of healing success, complications, and amputation rates by systematically assessing ulcer characteristics and stratifying patients based on SINBAD scores.

## Materials and methods

This prospective observational study was conducted over one year, from February 2024 to February 2025, at the Department of General Surgery, Krishnanagar Institute of Medical Science, Krishnanagar, India. Ethical approval was obtained from the institute's Institutional Ethics Committee before initiating the study, with approval number KIMS/IEC/2024/256. The study included 130 patients with T2DM and DFUs attending the outpatient department through a consecutive sampling approach.

Inclusion criteria

Included were patients diagnosed with T2DM, aged ≥18 years, with the presence of DFU confirmed clinically, and who consented to participate in the study.

Exclusion criteria

Patients with type 1 diabetes mellitus (T1DM), patients with ulcers due to non-diabetic causes (venous, arterial, traumatic), patients with the presence of systemic diseases like malignancy, severe renal failure, or immunodeficiency, pregnancy or lactating patients, and patients with known psychiatric illnesses or cognitive impairment that may affect compliance were excluded in the study.

Data collection

The patient's demographic details, duration of diabetes, and glycemic control (as measured by HbA1c) were recorded. The clinical examination of the ulcer involved assessing its site, size (measured in centimeters by length and width), depth, signs of infection, and vascular status using Doppler ultrasound. The SINBAD classification system was used to systematically score each ulcer at baseline and during follow-up visits. The SINBAD scoring system is shown in Table 1.

**Table 1:** SINBAD scoring SINBAD: Site, Ischemia, Neuropathy, Bacterial infection, Area, and Depth Table adapted from Ince et al. [12].

Items	Definition	SINBAD score
Site	Forefoot: distal to the tarsometatarsal joint	0
	Midfoot or hindfoot	1
Ischemia	Pedal blood flow intact: at least one pulse palpable	0
	Clinical evidence of reduced pedal blood flow	1
Neuropathy	Protective sensation intact	0
	Protective sensation lost	1
Bacterial infection	None	0
	Present	1
Area	Ulcer <1 cm²	0
	Ulcer >1 cm²	1
Depth	Ulcer confined to the skin and subcutaneous tissue	0
	Ulcer reaching the muscle, tendon, or deeper tissue	1
Total possible score		6

Based on their SINBAD scores, patients were stratified into three groups: low-risk group: score 0-2; moderate-risk group: score 3-4; and high-risk group: score 5-6. This categorization was used to analyze associations between SINBAD scores and clinical outcomes such as ulcer healing, need for minor or major amputation, and duration of hospital stay. While treatment decisions were made based on comprehensive clinical evaluation and standard care protocols, the SINBAD score served as a validated classification tool for comparing outcomes across patient groups.

Patients received individualized surgical management based on their SINBAD scores. Standardized surgical interventions included wound debridement, infection control measures, and reconstructive procedures such as grafting or flap coverage when indicated. Appropriate antibiotic therapy was administered based on microbiological culture results.

Follow-up

Patients underwent weekly follow-ups for the first month and then monthly follow-ups for up to six months thereafter. During follow-up visits, the progress of ulcer healing, re-ulceration, and new ulcer development, as well as limb salvage success, amputation rates, and complications such as infections, were documented.

Outcome measures

The primary outcomes of the study included ulcer healing rates, limb salvage rates, and amputation rates. Ulcer healing was defined as complete epithelialization of the wound without discharge or infection within the six-month follow-up period. Limb salvage was evaluated by the avoidance of major amputation (defined as amputation above the ankle level). Amputation rates, including both minor (toe or forefoot) and major amputations, were recorded and correlated with SINBAD scores to assess predictive accuracy.

Data analysis

The data were collected, entered, and analyzed using IBM SPSS Statistics for Windows, Version 29.0 (IBM Corp., Armonk, New York, United States). Continuous variables were expressed as mean±standard deviation, while categorical variables were presented as frequencies and percentages. The relationship between SINBAD scores and primary outcomes such as ulcer healing, limb salvage, and amputation rates was analyzed using the chi-squared test. A p-value of <0.05 was considered statistically significant. 

## Results

This study included a total of 130 patients with T2DM and DFUs. The demographics and clinical characteristics of the patients are shown in Table 2. The mean age of the patients was 62.65±12.5 years, and the majority of the patients were in the 61-70-year age group, accounting for 74 (56.9%). A male preponderance was observed in this study, comprising 88 (67.7%) of the participants. Common comorbidities observed were hypertension (61, 46.9%), dyslipidemia (45, 34.6%), cardiovascular disease (35, 26.1%), and chronic kidney disease (20, 15.4%). The mean duration of diabetes was 12.54±3.12 years, and the average HbA1c level was 9.32±2.65%, indicating suboptimal glycemic control.

**Table 2:** Demographics and clinical characteristics of the patients The data are presented in N (frequency) and % (percentage) and mean±SD.

Parameters	Values (n=130)
Age in years (mean±SD)	62.65±12.5
Age distribution (years), N (%)	
40-50	11 (8.5%)
51-60	35 (26.9%)
61-70	74 (56.9%)
>70	10 (7.7%)
Gender, N (%)	
Male	88 (67.7%)
Female	42 (32.3%)
Comorbidities, N (%)	
Hypertension	61 (46.9%)
Dyslipidemia	45 (34.6%)
Chronic kidney disease	20 (15.4%)
Cardiovascular disease	34 (26.1%)
Duration of diabetes in years (mean±SD)	12.54±3.12
HbA1C in % (mean±SD)	9.32±2.65

The characteristics of DFUs are shown in Table 3. The major foot ulcer site was the forefoot in 112 (86.1%) of the cases. Ischemia was present in 46 (35.4%), neuropathy in 85 (65.4%), and bacterial infection in 119 (91.5%) of the patients, respectively. The majority of patients had an ulcer size greater than 1 cm² in 124 (95.4%) and a superficial ulcer in 82 (63%), respectively.

**Table 3:** Ulcer characteristics of the patients The data are presented in N (frequency) and % (percentage).

Parameter	Total no. of patients (n=130)
Foot ulcer site, N (%)	
Forefoot	112 (86.1%)
Midfoot	6 (4.6%)
Hindfoot	12 (9.2%)
Ischemia, N (%)	46 (35.4%)
Neuropathy, N (%)	85 (65.4%)
Bacterial infection, N (%)	119 (91.5%)
Size of the ulcer, N (%)	
<1 cm^2^	6 (4.6%)
>1 cm^2^	124 (95.4%)
Ulcer depth, N (%)	
Deep	48 (37%)
Superficial	82 (63%)

The distribution of the SINBAD scores is shown in Table 4. In this study of 130 patients, the majority had a SINBAD score of 3, 72 (55.4%), indicating moderate ulcer severity. Scores of 1 and 2 accounted for 10 (7.7%) and 15 (11.5%) of patients, respectively, representing less severe ulcers. Higher scores of 4, 5, and 6 were less common, observed in 27 (20.8%), four (3.1%), and two (1.5%) of patients, respectively, reflecting fewer cases with severe ulcer characteristics.

**Table 4:** Distribution of SINBAD scores among the patients SINBAD: Site, Ischemia, Neuropathy, Bacterial infection, Area, and Depth The data are presented in N (frequency) and % (percentage).

SINBAD score	N (%) (n=130)
1	10 (7.7%)
2	15 (11.5%)
3	72 (55.4%)
4	27 (20.8%)
5	4 (3.1%)
6	2 (1.5%)

In the present study, patients with a SINBAD score of 1 achieved 90% healing without any amputations, while none underwent major amputations. The incidence of major amputations was higher in grade 6, two (100%), compared to grade 5, three (75%), grade 4, eight (29.6%), and grade 3, five (6.9%), respectively. Overall, this association was found to be significant (p<0.05). The results are shown in Table 5.

**Table 5:** Association between SINBAD score and clinical outcome SINBAD: Site, Ischemia, Neuropathy, Bacterial infection, Area, and Depth The data are presented in N (frequency) and % (percentage). * indicates significance (p<0.05) (chi-squared test).

SINBAD score	Major amputations N (%)	Nonhealing N (%)	Healing/healed N (%)	Total (N)	Chi-square (X^2^) and p-value
1	0 (0%)	1 (10%)	9 (90%)	10	X^2^=42.15; p<0.001*
2	1 (6.7%)	2 (13.3%)	12 (80%)	15	
3	5 (6.9%)	27 (37.5%)	40 (55.6%)	72	
4	8 (29.6%)	14 (51.9%)	5 (18.5%)	27	
5	3 (75%)	1 (25%)	0 (0%)	4	
6	2 (100%)	0 (0%)	0 (0%)	2	
Total	19 (14.6%)	45 (34.6%)	66 (50.8%)	130	

Table 6 demonstrates a significant association between SINBAD scores and amputation outcomes (p=0.001). Patients with low SINBAD scores (1-2) predominantly avoided amputation, with 10 (100%) and 12 (80%) limb salvage, respectively. Higher scores (5-6) were strongly associated with major amputations, reaching rates of 3 (75%) and 2 (100%). This highlights the effectiveness of the SINBAD score in predicting amputation risk in DFUs.

**Table 6:** Association between SINBAD score and amputation outcome SINBAD: Site, Ischemia, Neuropathy, Bacterial infection, Area, and Depth The data are presented in N (frequency) and % (percentage). * indicates significance (p<0.05) (chi-squared test).

SINBAD score	No amputation N (%)	Minor amputation N (%)	Major amputation N (%)	Total (N)	Chi-squared (X^2^) and p-value
1	10 (100%)	0 (0%)	0 (0%)	10	X^2^=25.87; p=0.001*
2	12 (80%)	2 (13.3%)	1 (6.7%)	15	
3	60 (83.3%)	8 (11.1%)	4 (5.6%)	72	
4	9 (33.4%)	10 (37%)	8 (29.6%)	27	
5	0 (0%)	1 (25%)	3 (75%)	4	
6	0 (0%)	0 (0%)	2 (100%)	2	
Total	91 (70%)	21 (16.2%)	18 (13.8%)	130	

## Discussion

DFUs represent a significant cause of morbidity and limb loss among patients with T2DM, significantly affecting quality of life and healthcare resources. Early and accurate evaluation of DFU severity is crucial for effective management and preventing complications such as amputation [13]. The SINBAD scoring system, which assesses six critical ulcer parameters, has emerged as a practical and reliable tool for risk stratification. This study focused on exploring the relationship between SINBAD scores and amputation outcomes in patients with T2DM [11]. By establishing this association, the study aims to support clinicians in making informed decisions to improve patient prognosis and reduce the burden of diabetic foot complications.

The study was conducted on 130 patients with T2DM and DFU, with a mean age of 62.65±12.5 years. The majority of patients are in the 61-70-year age group, accounting for 56.9%. This age distribution is consistent with the typical demographic affected by diabetic foot complications, as advanced age is a known risk factor [14]. Likewise, in a study done by Piran et al., the mean age of the T2DM patients with DFU is 63.34±13.22 years. The study demonstrated a male predominance, with 67.7% of participants being male [15]. This aligns with existing literature suggesting males are at higher risk for DFUs, possibly due to differences in occupational exposure, footwear habits, high pressure on lower limbs, and healthcare-seeking behavior [16]. Our results are in line with the previous report by Ha Van et al., which found that 75.8% of the DFU cases were males [9].

In this study, the forefoot was the predominant site of ulcers, accounting for 86.1% of cases, which aligns with the biomechanical stresses and pressure points common in diabetic neuropathic feet [17]. Ischemia was present in 35.4% of patients, indicating a substantial burden of peripheral arterial disease that impairs blood flow and delays wound healing, contributing to worse clinical outcomes [18]. Neuropathy was observed in 65.4% of patients, underscoring its critical role in ulcer formation due to the loss of protective sensation that increases the risk of unnoticed trauma. A striking 91.5% of patients had bacterial infections, highlighting the susceptibility of DFUs to colonization and the subsequent challenges in managing infection-related complications. Similarly, a study by Singh et al. [19] found that 68% of patients with DFU had bacterial infections. Most ulcers were larger than 1 cm² (95.4%), which is clinically significant as larger wounds tend to have prolonged healing times and higher rates of complications, including infection and amputation. While 63% of ulcers were classified as superficial, limited to the skin and subcutaneous tissues, they still necessitate prompt and diligent care to prevent deeper tissue involvement and adverse outcomes. Likewise, in a study by Shukla and Mishra [20], 68% of the DFUs are located in the forefoot region, with ischemia and neuropathy affecting 30% and 32% of cases, respectively, and bacterial infection in 74% and 94% of cases, with an area greater than 2 cm².

The distribution of SINBAD scores in this study reveals that most patients (55.4%) presented with a moderate ulcer severity, as indicated by a score of 3. This suggests that over half of the study population had ulcers, characterized by a balanced combination of factors such as site, ischemia, neuropathy, infection, area, and depth, placing them in an intermediate risk category. Lower scores (1 and 2), representing less severe ulcers, were less frequent, indicating that only a smaller portion of patients had relatively mild ulcer presentations. Conversely, higher SINBAD scores (4, 5, and 6), which denote more severe ulcer characteristics and increased risk of complications, were comparatively rare. This distribution highlights the varied clinical spectrum of DFUs in the study population and illustrates the value of using a standardized scoring system, such as SINBAD, to categorize ulcer severity for prognosis and management planning effectively. In a study conducted by Jayalal et al., most patients had a SINBAD score of 2, which encompasses 58%, indicating mild disease severity. Moderate DFU severity (scores 3 and 4) is observed in 11.3% and 21.3% of the patients [21]. In another study by Mitra et al., 20.5% of the patients had a SINBAD score >3, thus reflecting a severe form of disease [22].

The present study results highlight that patients with a low SINBAD score (grade 1) have a significantly higher chance of healing, indicating less severe ulceration and a better overall prognosis. Conversely, the highest SINBAD grade (6) is strongly associated with major amputation, reflecting advanced tissue damage and poor healing potential. This reinforces the reliability of the SINBAD score as a predictive tool for clinical outcomes in DFUs. Recognizing patients with severe ulcers early can help prioritize aggressive management to prevent limb loss. Overall, these findings support the integration of SINBAD scoring in routine clinical assessment for better treatment planning. Likewise, in a study conducted by Suruthi et al., the healing rate was higher in grade 1 (100%) and lower in grade 6, at 5.2% [23].

In the present study, higher SINBAD scores (5-6) were closely linked to an increased risk of major amputations, with rates as high as 75% and 100%, respectively. This indicates that severe ulcer characteristics, including extensive tissue involvement and poor vascular status, significantly compromise limb viability. The strong association underscores the importance of early identification and aggressive intervention for high-risk patients. These findings validate the SINBAD score as a reliable prognostic tool for predicting the risk of major amputation. Prompt management guided by this scoring can improve limb salvage outcomes. Similarly, in a study done by Gunawan et al., the incidence of amputation in DFU patients was higher in SINBAD grades 5 and 6, which encompass 33.8% and 22.8%, respectively [24]. Studies also indicate that a median follow-up of DFU patients for eight years showed that the SINBAD score is a significant predictor of mortality [25]. In a recent study done by Sridhar et al., the SINBAD score with a cut-off value of 3.5 is a reliable score to predict amputation with a sensitivity and specificity of 96.3% and 92.4%, respectively [26]. 

While the SINBAD scoring system proved useful in stratifying risk and predicting clinical outcomes, it does not account for all variables influencing DFU prognosis. For example, factors such as nutritional status, socioeconomic conditions, and access to multidisciplinary care may also impact healing but are not captured in the score. Additionally, inter-observer variability in the clinical assessment of parameters like ischemia or infection could affect score accuracy. Although our statistical models confirmed SINBAD as an independent predictor, its integration with other clinical tools or biomarkers could further enhance predictive value. Therefore, while SINBAD is practical and easy to implement, its use should be complemented by a comprehensive clinical evaluation.

The study limitations included being a single-center study, which may limit the generalizability of the findings to other populations. The sample size, although adequate, was relatively small for subgroup analyses. Additionally, factors such as patient adherence, socioeconomic status, and long-term outcomes were not fully explored.

## Conclusions

This study demonstrated that the SINBAD scoring system effectively stratifies DFUs according to severity, with higher scores strongly associated with increased rates of major amputations and poor healing outcomes. Patients with low SINBAD scores showed high healing rates and minimal need for amputation, while those with scores of 5 and 6 faced significantly greater risk of major limb loss. The significant correlation between SINBAD scores and clinical outcomes underscores the value of SINBAD as a practical prognostic tool. Incorporating SINBAD scoring into routine assessment can enhance the early identification of high-risk ulcers, enabling targeted interventions that improve healing, reduce amputation rates, and ultimately enhance patient quality of life.
